# Immunomodulatory Effects of Natural Feed Additives for Meat Chickens

**DOI:** 10.3390/life13061287

**Published:** 2023-05-30

**Authors:** Clive J. C. Phillips, Babak Hosseintabar-Ghasemabad, Ivan F. Gorlov, Marina I. Slozhenkina, Aleksandr A. Mosolov, Alireza Seidavi

**Affiliations:** 1 Institute of Veterinary Medicine and Animal Sciences, Estonian University of Life Sciences, Kreutzwaldi 1, 51014 Tartu, Estonia; 2 Curtin University Sustainability Policy (CUSP) Institute, Curtin University, Kent St., Bentley 6102, Australia; 3 Department of Animal Science, Faculty of Agriculture, University of Tabriz, Tabriz 5166616471, Iran; b.ht@tabrizu.ac.ir; 4 Volga Region Research Institute of Manufacture and Processing of Meat and Milk Production, 400131 Volgograd, Russia; niimmp@mail.ru (I.F.G.); slozhenkina@mail.ru (M.I.S.); a-leksha@mail.ru (A.A.M.); 5 Department of Animal Science, Rasht Branch, Islamic Azad University, Rasht 41335-3516, Iran

**Keywords:** poultry, broilers, immune system, natural feed additive, antibiotic alternative

## Abstract

Broiler chickens are increasingly kept in large numbers in intensive housing conditions that are stressful, potentially depleting the immune system. With the prohibition of the use of antibiotics in poultry feed spreading worldwide, it is necessary to consider the role of natural feed additives and antibiotic alternatives to stimulate the chickens’ immune systems. We review the literature to describe phytogenic feed additives that have immunomodulatory benefits in broilers. We initially review the major active ingredients from plants, particularly flavonoids, resveratrol and humic acid, and then describe the major herbs, spices, and other plants and their byproducts that have immunomodulatory effects. The research reviewed demonstrates the effectiveness of many natural feed additives in improving the avian immune system and therefore broiler health. However, some, and perhaps all, additives have the potential to reduce immunocompetence if given in excessive amounts. Sometimes additives are more effective when given in combination. There is an urgent need to determine tolerance levels and optimum doses for additives deemed most suitable to replace antibiotics in the diet of broiler chickens. Effective replacement is most likely with readily available additives, such as olive oil byproducts, olive leaves and alfalfa. It is concluded that effective replacement of antibiotic function with plant-derived additives will be possible, but that further research is necessary to determine optimum doses.

## 1. Introduction 

**Figure 1:**
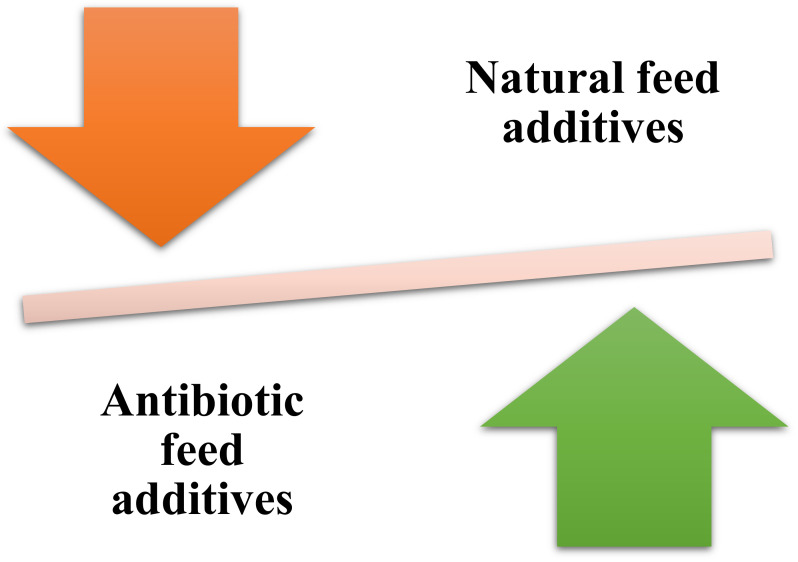
The use of natural feed additives in poultry nutrition is increasing as the use of antibiotics is declining.

Increasing demand for poultry products and a growing concern over the widespread use of antibiotics in poultry diets have supported the search for natural feed additives that can replace antibiotics in sustaining a high meat output [1]. At the same time, the disease threats to poultry are increasing as the industry intensifies, with greater stress on the birds and larger populations, both of which facilitate disease spread [1]. Antibiotics have been used mostly as growth promoters, though they may also be used as prophylactic agents and therapeutic agents [2]. Resistance conferred to bacteria targeted by antibiotics routinely included in poultry feed has been passed to humans, which presents one of the major challenges to controlling human disease in the 21st century [1]. As a result, governments worldwide, but particularly in Europe, have started to ban the use of antibiotics in poultry feed. With widespread bans and restrictions on the use of antibiotic growth stimulants, the focus of research has shifted to finding other additives that can acceptably lead to disease prevention, generation of microbiota, adjustment of the intestinal microbial ecosystem, and finally to strengthening and maintaining the health of the host through improving immunity (Figure 1) [3,4].

In place of antibiotics, feed additives are being assessed for their ability to enhance broiler production, and particularly for their ability to enhance the avian immune system, enabling them to naturally control infection. Feed additives have been defined as ‘low inclusion products used in diet formulations for purposes of improving the nutritional quality of feed or the animal performance and health’ [5]. However, the European Union defines feed additives more broadly in EC regulation 1831/2003 [6] as ‘substances, micro-organisms or preparations, other than feed material and pre-mixtures which are intentionally added to feed or water to favourably influence *inter alia* the (i) characteristics of feed or animal products, (ii) environmental consequences of animal production, (iii) performance, health or welfare through their influence on gut microflora profile or feed digestibility, or (iv) to have a coccidiostatic or histomonostatic effect.’ In human nutrition, food additives may be defined even more broadly as substances not normally found in foods [7]. 

Additives used in poultry feed to replace antibiotics include probiotics, prebiotics, organic acids, such as propionic acid to reduce gut pH, antimicrobials such as lactoferrin that directly inhibit the growth of harmful bacteria, and finally, phytogenic additives, which include natural substances derived from plants: herbs, spices, plant extracts and essential oils [5]. Spices are made from non-herbaceous parts of plants, in particular seeds and roots, usually dried and crushed. Herbs are made from herbaceous parts and are often used fresh [8]. Some plants, such as garlic, are hard to classify. Natural feed additives have received the least attention in animal nutrition [9], despite their being relatively effective and inexpensive. In human nutrition, they have been recognized for millennia for their beneficial effects on health [10,11].

As in other animals, immunity is conferred in poultry both passively and actively. These two arms of the immune system complement each other and work together to protect the host. Acquired immune systems allow the innate immune system to respond quickly to new pathogens, but the mechanisms it uses can be metabolically and physiologically costly for the host [12,13]. Heterophils and cytokines are particularly relevant to monitor for poultry immune status [14].

Many nutrients are able to modulate the immune system. The concept of nutritional safety modification goes beyond functional disorders associated with levels of deficiency or toxicity of various nutrients and involves the use of specific nutrients to achieve a sustainable functional goal. Nutritional safety modification can be defined as the targeted supplementation of specific dietary nutrients to alter some aspects of immune function to achieve the desired goal [12]. The use of diet to change the function of the immune system in a production environment without the use of antibiotics has become particularly important. Deficiencies of several nutrients can reduce immune system function, including dietary protein, lysine, arginine, methionine, and vitamins D and E. 

This review considers the application of natural feed additives in the poultry sector to improve immunity in birds, assisting them to maintain growth rates comparable with those achieved with the inclusion of antibiotics in their feed and enabling them to be healthy and disease-free throughout their lives. For poultry, there are many plant products that have antimicrobial and immunomodulatory properties, but here we review mainly the most common herbs, spices, other plants and plant byproducts that have the potential to be utilised on a large scale to replace antibiotics in feed (Table 1). We did not extensively review the mode of action of the various potentially efficacious compounds or plant-derived feed additives, because of the considerable expansion to the scope of the paper that this would entail. However, we aimed to review the evidence for the efficacy of the major potential plant-derived alternatives to antibiotics. Literature sources were sought from the Web of Science and, where necessary, other search engines. 

## 2. Beneficial Compounds in Plant Extracts

A series of plant compounds are extracted from fruits and their byproducts. One of the most beneficial are the flavonoids, which can be used as phytogenic additives in poultry nutrition [15]. The bioactive compounds of this category of additives include catechin, epicatechin, gallium acids, gallocatechin, and epigallocatechin, which have significant antimicrobial effects against some pathogens associated with poultry [14]. Gallic acid, one of the most common phenolic compounds in plants, has been widely studied and has beneficial effects in the treatment of a wide range of diseases [14,16]. Ellagic acid is the dimerized form of gallic acid, and it inhibits aflatoxin production by fungal species in many berries [14]. It can also reduce the symptoms of colitis in mice and inhibit the growth of several types of bacteria, such as *Helicobacter pylori* [14]. Anthocyanins are a type of flavonoid found in fruits such as berries, grapes and strawberries [14]. 

There are more than 4000 flavonoids in plants, most of which are valuable bioactive compounds that can play an important role as health and immunity boosters [17,18]. Prominent plants with significant amounts of flavonoids that could play a role in improving immunity in poultry nutrition include chamomile, dandelion, ginkgo, green tea, hawthorn, licorice, passionflower, milk thistle, onions, rosemary, sage, thyme, and yarrow. Flavonoids have extensive biological properties that have generally been found to improve animal health and help reduce the risk of disease [17,19]. However, economic benefits in poultry systems have yet to be reliably demonstrated. There is evidence that they improve immunoglobulins in chickens, reduce caecal coliforms and protect birds from a range of diseases, particularly when they are under heat stress. They have also been shown to increase the vitamin C levels in quail [20]. Improvements in the immune status of geese have also been observed [20,21]. Such benefits have been found with feed additives such as alfalfa, cranberry extract, citrus byproducts and green tea extract. These additives contain more than just flavonoids, but they are believed to be the main active ingredient. 

Flavonoids such as quercetin and pomegranate fruit polyphenols induce regulatory T cells by inhibiting mTOR (mechanistic target of rapamycin), at least in human cancer cells, and reducing inflammatory markers [14,22]. Quercetin is the most common flavonoid in nature [23,24]. Wild birds (*Sylvia atricapilla*), the ancestors of today’s industrial and farmed poultry strains, are reported to choose their food depending on the perceived flavonoid content [25]. Flavonoids are also found in fruits and vegetables, such as olives, onions, cabbage and apples. The bioactive contents of pulp and fruit extracts can destroy free radicals, thereby preventing disease [14].

Natural antioxidants in the diet (vitamin E, carotenoids, selenium, etc.), antioxidants synthesized in the body (glutathione, thioredoxin, antioxidant enzymes, etc.) and the balance between antioxidants and prooxidants in cells, are all important for maintaining a high level of immunity in poultry [26,27]. Antioxidants in these two groups, natural and artificial, are a major focus in poultry nutrition since reactive oxygen species are generated by a diet rich in polyunsaturated fatty acids. Although the artificial antioxidants hydroxyanisole butylated, hydroxytoluene butylated and propyl gallate have been commonly used to reduce peroxidation in feed, there may be adverse public acceptance of artificial additives, compared to natural antioxidant ones [28]. Natural antioxidants, such as those in the calyx and seed of the persimmon plant, can therefore play a greater role in improving the immune system function since they minimize side effects and reduce consumer concern [29]. Vitamin E analogs, which include various types of tocopherols and tocotrienols, are a source of strong antioxidants that can perform several physiological functions, such as the regulation of metabolic processes and the improvement of anti-inflammatory and anti-cancer properties, thereby strengthening the immune system. However, many of these functions can be replaced by natural antioxidants, which may even have greater activity than the artificial ones [30]. A particularly valuable natural antioxidant group is the carotenoids, which protect fats from oxidative damage by deactivating dioxidin (without decomposition) and reacting with hydroxyl, superoxide and peroxyl radicals. Compared to phenolic compounds and other antioxidants, carotenoids are not just specific neutralizers of peroxyl radicals, they increase bactericidal activity [31]. Carotenoids are so important in these roles in poultry nutrition that genetically-engineered corn with enhanced carotenoids has been produced [32]. 

A large range of plants have antioxidant properties: black seed, ginger, *Artemisia annua*, red hot pepper, thyme, rosemary, dill, chicory, radix bupleuri, *Moringa olifeira*, *Scutellaria baicallensis*, Java turmeric (*Curcuma xanthorrhiza*), coriander (*Coriandrum sativum*)cinnamon, liquorice, *Ginkgo billoba* and plants containing resveratrol (red grapes, blueberries etc) [33]. Isoflavones (ISFs), such as daidzein, genistein, and glycitein, have significant benefits to host health because of their antioxidant properties and ability to strengthen the body’s immune system [24]. The flavones have antioxidant, antibacterial, antiviral, neuroprotective and hepatoprotective benefits. They are particularly effective against infectious bursal disease virus in broilers, reducing both bursal lesions and viral protein expression [24,34]. Plasma antioxidant activity can be achieved in broilers by 40–80 mg ISF/kg in the diet [24,35]. 

Resveratrol is a natural polyphenolic compound found mainly in grapes, *Polygonum cuspidatum* and peanut [36]. The use of 400 g/kg of resveratrol has been found to promote intestinal development and antioxidant activity in the intestine, via the expression of nuclear factor erythroid 2-related factor 2 (Nrf2), an important transcription factor in the antioxidant system [37]. This is particularly important during heat stress, which causes oxidative damage to the intestinal mucosa and the production of large numbers of reactive oxygen species [37]. Resveratrol also has anti-inflammatory, anti-oxidation and energy metabolism regulating properties [38,39], which leads to improved immunity and the maintenance of bird health [24,36]. It increases *Lactobacillus*, *Bifidobacterium*, *Bacteroidetes*, *Akkermansia* and *Ruminococcus* spp and reduces *Lactococcus*, *Clostridium*, *Oscillibacter*, *Hydrogenoanaerobacterium* species, with tight junctions strengthened and reduced LPS permeability [24,40,41].

Although not from living plants, humic acid is a complex organic acid formed naturally from the decomposition of organic matter and coal [42]. It forms a thin protective layer in the intestine, which inhibits pathogens and moulds [42]. It can have immune-stimulating, anti-inflammatory and antiviral properties, which increase the phagocytic activity of leukocytes, and activate neutrophils [43,44,45]. Consumption of 0.3% humic acid increases colonization by *Lactobacillus* and *Bifidobacterium* and modifies the immune system [44]. Despite the ability of *Bifidobacterium* to stimulate antibody production, it is also well-established that *Lactobacillus* is an immunomodulator due to its ability to reduce bacterial transmission in animals [46]. The improvement of the immune response with humate is dependent on its ability to activate neutrophils and macrophages [43,44].

## 3. Herbal Feed Additives

The most promising herbs that could be used as replacements for antibiotics include dill, oregano, ginkgo, sage, fennel, olive leaves, hogweed, coneflower, savory and some Chinese medicinal herbs. 

The dill plant (*Anethum graveolens* L.) from the *Apiaceae* family contains glycosides, saponins, tannins, terpenoids, steroids, flavonoids, phlobatanin, cardiac glycoside, anthraquinone, gallic acid, catechin, chlorogenic acid, luteolin and epicatechin. It has valuable health benefits [47,48], being able to reduce coliforms and increase lactobacilli in the caecum and jejunum [4]. Feeding 0.4 and 0.6% dill in the diet of broiler chicks has the potential to improve performance, reduce cholesterol and triglycerides, and improve microbial flora [49]. Oregano (*Origanum compactum*) is also potentially very useful, with carvacrol as one of the major active ingredients. It is effective against *Escherichia coli* and salmonella species in the caecum of broilers [4]. Another major active ingredient is thymol, which has similar effects [4]. 

Skullcap *(Scutellaria baicalensis*)*,* a Chinese herbal medicine, is an additive that has attracted considerable attention [33]. Flavones and other antioxidants can be extracted from the roots, with the main active compounds being wogonoside and aglycones baicalin [50]. In low doses, the main benefit of this herbal additive is in reducing heat and other stresses [33]. However, the inclusion of *Scutellaria baicalensis* root (SBR) at levels of 1 and 1.5% in poultry diets has been observed to reduce the size of the spleen and Bursa of Fabricius, as well as reducing lymphocytes and eosinophils [51]. 

Another common Chinese medicine herb, *Ginkgo biloba* (GB), has antioxidant, anti-apoptotic, anti-diabetic, anti-asthmatic, neuroprotective, cardiac protective and antitoxic benefits [52]. Fermented Ginkgo biloba probiotics (FGB) included at 0.2 and 0.4% in the diet can improve the growth and immunity of broilers while suppressing the pathogenicity of *Escherichia coli* [53]. The addition of *Ginkgo biloba* (0.06%) and peppermint (0.2%) in feeding broiler chickens under heat stress can improve the efficiency of feed conversion and increase the immune response against Newcastle Disease and infectious bronchitis viruses [54]. Feeding both Bacillus coagulans-fermented *Ginkgo biloba* (FG) and nonfermented *Ginkgo biloba* (NFG) forms to broiler chickens at 0.3% of the diet can affect Newcastle disease vaccine potency. The FG form increased the expression of antimicrobial defensin RNA, while the NFG form inhibited this vaccine [55].

Sage (*Salvia officinalis* L) is a commonly used Mediterranean herb, which has some control of *Escherichia coli* in chickens [56]. It has also led to increased eosinophil, monocyte and, heterophil and immunity titers against Newcastle disease and avian influenza viruses, at concentrations in the diet of 0.5 to 1.2% [57]. The effects of sage extract on immune parameters and antibacterial activity in broilers are promising. 

Fennel is another Mediterranean herb, whose extract has improved the effectiveness of vaccination against Newcastle Disease and increased immunoglobulin production [58]. In Safaei-Cherehh et al.’s study [58] fennel extract also improved resistance to infectious bronchitis virus, but it reduced it to infectious bursal disease. 

The olive tree (*Olea europaea* L.) is one of the most relevant Mediterranean phytogenics, and olive leaf extract has anti-hypertensive, anti-atherogenic, anti-inflammatory, blood sugar lowering, and blood cholesterol lowering properties [24]. These extracts contain many potentially bioactive compounds, especially phenolic acids, phenolic alcohols (hydroxytyrosol), flavonoids, and secoiridoids (oleuropein) [24,59]. The phenolic compounds of olive oil help by positively regulating the expression of genes involved in maintaining tight junctions between intestinal cells, modulating the oxidative state of the intestinal epithelial layer, inflammatory and immune response, and maintaining the integrity of the intestinal barrier [60,61]. In addition, phenolic compounds extracted from olive leaves may be beneficial for broilers as antioxidants and through their antimicrobial activity against intestinal pathogenic bacteria [24,62]. Care has to be taken as leaves can have a high copper content from copper fungicides, which in the long term can damage the liver of consumers. About 25% of the leaf is oleuropein, which has antioxidative, antimicrobial and antiviral properties, and functions in the leaf to protect against insects and UV light [63]. Olive tree leaves also have a much higher phenol content than olive oil, with greater antioxidant potential [63]. Supplementation with olive leaf extract up to 25 mg/kg improved epithelial barrier function in experimental colitis models, as demonstrated by increased expression of mucin MUC-2, tight junction protein ZO-1 and TFF-3 [64]. The intestinal anti-inflammatory activity of olive leaf extract in murine colitis models may be related to its immunomodulatory properties and capacity to restore the intestinal epithelial barrier [24]. 

Hogweed (*Heracleum persicum*) is a plant in the carrot family, whose extract has antioxidant, anticonvulsant, analgesic, anti-inflammatory and immunomodulatory activities. These are attributed to a wide range of phytochemical compounds, including volatile compounds, terpenoids, triterpenes, furanocoumarins, flavonoids and alkaloids [65,66]. A *Heracleum persicum* supplement at levels between 1 and 2.5 ml/L can improve the function of the immune system in broiler chickens [66]. Additionally, feeding levels of 0.5 and 0.75% hogweed led to an increase in the titer against avian influenza and the microbial population of *Lactobacillus* and a decrease in *Escherichia coli* in the ileum [67]. When included at levels of 100–200 mg/L, the extract of this plant increases the antibody titer of broilers against Newcastle disease virus [68]. The reason for these health-giving properties could be the presence of flavonoids or furanocoumarins in the *H. persicum* plant, leading to an increase in humoral immunity. *Heracleum persicum* extract can also stimulate macrophages and beta lymphocytes that play a role in antibody synthesis.

The purple coneflower (*Echinacea purpura*) can increase lymphocytes and reduce heterophils, in conjunction with an antibiotic, probiotic, organic acid, and vitamin C [69]. Summer savory (*Satureja hortensis* L.) and its extract have widespread antimicrobial properties and can reduce the concentration of *Escherichia coli* in the digestive tract [70].

The thyme plant (*Thymus vulgaris*) increases growth in broiler chickens, but there is some evidence that it does not improve immunity [71]. In support of this, Belali et al. found no effect of thyme extract on broiler immunity, but it did increase the growth rate [72]. However, there are many other reports of improved immune function with the addition of thyme or thyme oil to broiler diets, e.g., Fallah and Mirzaei found that it improved antibody titers to Newcastle disease and influenza [73]. 

The chicory plant (*Cicorium intybus* L. in the *Asteraceae* family), considered by most to be a herb, contains fructooligosaccharides, inulin, coumarins, and flavonoids [74]. When added to the diet of broilers it improves the immune system in the ileum, which has more lactobacilli and reduced *Escherichia coli* populations [74]. Similar benefits have been attributed to pennyroyal (*Mentha pulegium* L.), although in the jejunum, rather than the ileum, and improved resistance to Newcastle disease has been recorded [4]. Yarrow (*Achillea millefolium var. occidentalis*) included at 0.5–1.5 g/kg in the diet has produced either no effect or increased lactobacilli and reduced *Escherichia coli* in the ileum. 

## 4. Feed Additives from Spices

Spices that could be used as alternatives to antibiotics include ginger, cinnamon, coriander, cumin, garlic and red and black pepper [15]. Ginger (*Zingiber officinale roscoe*) contains several active compounds including gingerol, shogaols, gingerdiol and gingerdione [4,75]. The oil extract from ginger contains a high proportion of sesquiterpenes (67%), monoterpenes (17%) and aliphatic ingredients (14%) [75]. It has a strong antioxidant, antimicrobial and antifungal activity in the gut, greater than that of turmeric [75,76]. However, when included at 0.5–1.5 g/kg in the diet it has produced mixed results on immunity, with high level doses destroying the gut microflora, but smaller doses having some control of pathogens in the gut [4]. Both ginger and garlic (*Allium sativum*) are active against *Escherichia coli*, though garlic has broader spectrum activity [77]. The mechanisms are enhancement of phagocytosis and bactericidal activity and reduction of NO production. Many other plants in the allium genus have antimicrobial properties, with more than 100 phytotherapeutic compounds, including alliin, allicin and allyl isothiocyanate [77]. 

Coriander (*Coriandrum sativum* L.) is both a herb and a spice. The seed and coriander extract contain several beneficial pharmaceuticals, which potentially have antibacterial, antioxidant, antidiabetic and hypolipidemic properties [78]: linalool (67.70%) and *α*-pinene (10.5%); *γ*-terpinene (9.0%); geranyl acetate (4.0%); camphor (3.0%); and geraniol (1.9%) [79]. It has been suggested that this alone can effectively replace synthetic antibiotics in the diet of poultry [80]. Improvement has been observed in the antibody titers against common diseases, such as Newcastle, infectious bronchitis, and bursal disease infections in birds receiving coriander extract in water (1000 and 1250 ppm of coriander extract and 2 and 2.5% coriander powder) [81] and also in birds receiving a combination of 2% coriander seeds and 0.5% black pepper [78]. 

A plant native to Iran and Turkmenistan known as galbanum (*Ferula gummosa boiss*), from the *Apiaceae* family, produces oleo gum resin. The resin potentially protects against gram-negative bacteria [82], with an increased immune response to Newcastle disease challenge and increased spleen weight when 1% galbanum was included in the diet of broilers. 

Sumac (*Rhus coriaria* L.) is a plant species belonging to the *Anacardiaceous* family, and its fruit contains active compounds of flavonols, phenolic acids, hydrolyzable tannins, anthocyanins and organic acids. Sumac seed powder (SSP) is produced by grinding dried fruits and has defensive benefits for many health-related problems, including reducing *Escherichia coli* and strengthening health [83,84]. 

Black cumin (*Nigella sativa* L.) contains bioactive compounds, nogelleone, thymoquinome, and thymohdroquinone, giving it anti-toxic and antimicrobial properties and increasing the defense mechanisms against infectious diseases [85]. At high doses, black cumin improved antibody titers against Newcastle Disease and it was particularly effective against salmonella species [86]. 

Fenugreek (*Trigonella foenum-graecum* L.) also increases antibody titers [87]. Fenugreek is more effective than rosemary at immunological control in broilers [88]. 

The clove plant, a common spice, is rich in eugenol, an antibacterial agent. In chickens, it has the ability to reduce the prevalence of pathogens in the intestine and increase the size of the spleen [89]. The chili pepper (genus *Capsicum*, family *Solanacea*) contains dietary capsaicin, which prevents high-fat diet-induced metabolic endotoxemia and chronic low-grade systemic inflammation by increasing cecal butyrogenic bacteria and consequently butyrate levels, inhibiting colonic cannabinoid receptor type 1 (CB1) and reducing LPS biosynthesis [90]. Therefore, capsaicin prevents intestinal dysbiosis and metabolic endotoxemia, which are associated with chronic inflammatory diseases, and it plays a role in improving immunity [24]. Capsaicin increases the ratio of Firmicutes/Bacteroidetes and the abundance of *Faecali bacterium.* In addition, it increases the plasma level of glucagon-like peptide-1s(GLP-1) and gastric inhibitory polypeptide (GIP), and decreases the plasma ghrelin level [24,90]. Poultry do not feel the heat of capsaicin in the same way as humans, due to the lack of specific receptors to bind capsaicin or the lack of capsaicin-sensitive receptors [24]. However, it stimulates the immune system. In broiler chickens, a supplement of 80 mg/kg of natural capsaicin extract in diets improves antioxidant status and immune function. Capsaicin extract decreased the serum concentration of TNF-α and IL-1β and increased the total antioxidant capacity of catalase, glutathione peroxidase, and superoxide dismutase [24,91].

## 5. Other Plants with Immunomodulatory Effects

Alfalfa (*Medicago sativa* L.) is an important source of various minerals and vitamins, flavonoids, phenolic acid, xanthophylls, and phytochemical compounds, such as alfa-carotene, beta-carotene, beta-sitosterol, chlorophyll, coumarin, cryptoxanthin, daidzein, fumaric acid, genistein, limonene, lutein, saponins, stigmasterol, and zeaxanthin [4,92,93]. Polysavone, a natural extract from alfalfa, increased the relative weight of the thymus and spleen and the weight of the bursa, T and B lymphocytes and serum antibody titer, as well as inhibiting anti-Newcastle disease virus hemagglutination [94]. Alfalfa ethanol extract (at 0.1–0.15 g/kg feed) would be a good alternative to antibiotics in poultry [95]. Similarly, research [95] has confirmed the benefit of alfalfa extract in increasing the total number of white blood cells (WBC) and the number of lymphocytes. It is probably the saponins in alfalfa that stimulate the immune system to produce a series of antigen-specific and non-specific immune responses, increase the permeability of the intestinal mucosa, and enhance the absorption of viral antigens [92].

Green tea (*Camellia sinensis*) has antimicrobial, antioxidant, and immune-modulatory therapeutic properties when fed to meat chickens. It improves hemorrhagic responses to influenza and Newcastle disease challenges, as well as reducing *Escherichia coli* and increasing lactobacilli in the ileum and caecum of broilers [4].

The inclusion of turnip extract in the diet of chickens can be an alternative to conventional antibiotics because turnip extract has antibacterial properties [96]. Coliforms and gram-negative bacteria decreased, demonstrating direct bactericidal effects, and immunity was strengthened, with increases in antibody production [96,97].

The addition of edible basidiomycete mushroom (*Agaricus bisporus*) at the rate of 5% in the diet of 49 d old broiler chickens increased body weight [98]. Inclusion at 3% led to an increase in the antibody titer against Newcastle disease (ND) and an increase in the antibody titer against sheep red blood cells (SRBC) [99]. Varying results for different types of mushrooms may be attributed to several factors, for example, the type and part of the plant used, the time of harvest, the methods of preparing plant additives, and the methods of extracting plant bioactive compounds [92,99,100]. 

Extracts of cat’s claw (*Dolichandra unguis cati*) plant stimulate T cells, macrophages and other components of the immune system [17]. The globe artichoke (*Cynara scolymus*, a member of the daisy family) increases the humoral immune response when added to the diet of broiler chickens [101]. Inclusion at 500 ppm gave a higher antibody titer to avian influenza than 250 ppm [102]. Another member of the daisy family, milk thistle (*Silybum marianum*) has considerable antioxidant properties [102]. It has protective effects against aflatoxin b1 in broilers, which can damage the liver [103], and contains the flavonoids silybin, silychristin, and silydianin, collectively called silymarin, which has the ability to absorb and neutralize oxygen free radicals [104]. Along with seaweed (*Spirulina platensis)* and binder toxin, milk thistle has the ability to improve immunological performance [105].

*Artemisia annua* is one of the most important species of the daisy family, renowned for its antimalarial properties. In addition, artemisinin, the active compound of this plant, can be effective against colon cancer and leukemia [106]. Baghban-Kanani et al. [107] reported that feeding *Artemisia annua* in the diet of laying hens increased GSH-Px concentration and decreased MDA. Previous research indicated that it improved the antioxidant status and immune system [108].

Milkvetch (*Astragalus* species) can increase antibody titer, and plasma IL-2 and IFN-ᵧ content in chickens [109]. The plant can improve the antioxidant, antiviral, antimicrobial and anti-parasitic status of poultry and act as an immune system stimulant [110]. Oral administration of the *Astragalus* plant can be a vaccine booster and immune regenerator for poultry [111], acting as a natural probiotic to stimulate the immune system and strengthen the intestinal microbiota. However, there are over 3000 *Astragalus* species and the efficacy of the most suitable of these needs to be compared. 

Arfaj (*Rhanterium epapposum)* and desert thorn *(Lycium shawii)* are both natives of the Arabian peninsular. After methanol extraction, both are reported to increase the immune responses of broilers [112]. This research suggested increased serum catalase (CAT) and superoxide dismutase (SOD) enzymes, as well as increases in cellular and humoral immune responses [112]. Supplementation of broiler diets with the algae spirulina (*Spirulina platensis*) increases antioxidant activity [113]. 

## 6. Immunomodulatory By-Product Feed Additives

Byproducts often have a lower processing cost compared to other additives, due to fewer purification steps [14]. The use of byproducts in poultry nutrition is likely to be economically more feasible than crops grown specifically to produce feed additives [114]. 

Fruit pomace contains a large number of compounds that can synergistically play an important role in modulating the immune system and digestive tract microflora [14]. Fruit pulp is a good source of flavonoids, as well as being rich in vitamins and minerals. As described previously, plant flavonoids can modulate the immune system by reducing the production of pro-inflammatory cytokines, T-cell activation and proliferation [14].

Mulberry pomace and ethanolic extract could be an ideal alternative to antibiotics to prevent and reduce diseases, even coccidiosis [14]. Orange pulp contains ascorbic acid compounds, phenolic compounds, coumarin and several volatile compounds [aldehydes, esters, terpenes, alcohols, ketones, carotenoids (beta-carotene lutein and beta-cryptoxanthin)], nobiletin, pectin and bioflavonoids (hesperidin, naringenin and hysteritine) [2]. Orange byproducts improve immunity in poultry [2]. Orange pulp in the diet of poultry improved the antioxidant status of the blood, as well as red and white blood cell concentration [115,116]. It can be included in up to 12% of the diet of laying hens with no negative effect on the birds’ health. The dry peel of sweet orange (*Citrus sinensis*) strengthens the immune system of broiler chickens [117], and increases the antibody titer response to sheep red blood cells (SRBC) as well as immunoglobulin G (IgG) and IgM. It also increases the number of white blood cells, heterophils, lymphocytes, and monocytes [118,119]. It contains ethyl acetate extract, which supports the growth of Gram-positive bacteria (*Staphylococcus aureus*, *Bacillus cereus*, and *Listeria monocytogenes*), yeasts, and at the same time prevents the growth of moulds and pathogens [119]. Olive pomace in the diet of chickens increases the antibody titer to infectious bronchitis and Gumboro disease [120]. Consumption of olive pulp decreased the weight of the thymus in broiler chickens [121].

Some non-vegetable byproducts can also play an effective role in improving the safety and health of poultry as feed additives, such as earthworm meal, which contains lectins, antimicrobial peptides, pore-forming proteins, phenol oxidases and proteases [122]. It has beneficial effects on both the systemic and humoral immunity of broiler chickens [123,124].

## 7. Availability and Feasibility of Replacing Antibiotics

As well as the efficacy of any replacement, it is important to consider the supply chain and the feasibility of the replacement. Some of the possible alternatives, such as sumac berry, would take some years to become available and a large area of dedicated production land. Others, such as supplementation with marigold flower leaves extract, are efficacious in terms of health improvements [62] but would require a major new industry to be developed. Some beneficial additives, such as residues from olive oil production, are essentially byproducts and are already produced on a large scale [125]. It is necessary to study the availability, price and suitability of individual additives in individual locations, recognising that varied feed additives may be effective in different regions of the world. About 25 million tonnes of olives are grown annually, most for table use, giving the potential for leaf harvesting as a by-product from the same trees. Most are grown in the southern Mediterranean countries, which are well placed to supply byproducts to the poultry industries of the world. Additionally, a by-product of olive oil production, olive pomace oil, which comes from second pressings of the pomace or low-quality fruit, is not safe for human consumption because of carcinogenicity risks but could be suitable for poultry because of its short lifespan. Olive pomace has a moisture content of approximately 70%, hence needing extensive separation and drying before it could be incorporated into chicken feed. This dewatering is usually done by squeezing through filters to produce olive filter cakes [125]. Pomace is also sold to be extracted with solvents such as methanol or hexane for secondary oil production [126]. The resultant waste, olive pulp, is used as an animal feed. Large quantities of olive mill wastewater are produced which are also rich in flavonoids and phenolic compounds, but these have to be separated to obtain the phenols and other beneficial compounds before incorporation into animal feed. The product currently represents a potential environmental pollutant [127]. It should be noted that there are competing markets for olive byproducts, in functional foods, biodegradable packaging, biogas production and the pharmaceutical industries. It must also be remembered that small doses of two or more additives may work better than large doses of a single additive [128].

There is an urgent need to move on from proving the efficacy of individual plant feed additives as replacements for antibiotics to comparing their potency and determining optimum inclusion rates, e.g., [88]. Finally, price comparisons and the cost–benefit equation for the different contenders for antibiotic replacement need to be investigated. In relation to this, poultry feed represents between 40 and 50% of the global feed market, at about 500 million tonnes for the combined broiler and layer markets [129]. A 1% inclusion of feed as antibiotic replacement represents a global annual requirement of about 5 million tonnes of additive. Some byproducts are currently produced in large quantities, for example, it is estimated that about 40 and 2.5 million tonnes of olive pomace and leaf waste, respectively, are produced annually, the latter mainly from annual pruning of the trees [63,130]. Currently fed to cattle, or burnt for extraction of the phenolic compounds, the byproducts could make a significant contribution to the worldwide requirements for antibiotic replacement in poultry diets. The stability of the additives also must be considered, with some rapidly degrading under farm conditions, leading to varying concentrations in feed [131]. 

## 8. Summary and Conclusions

The feeding of natural compounds from medicinal plants, in the form of phytogenics, plant compounds extracted from fruits and other byproducts can improve the broiler chicken’s immune system. Several natural compounds are effective alternatives to antibiotics and meet the expectations of the poultry industry as a feed additive with the least risk and environmental pressure. We suggest that the most likely replacements on a large scale are fruit industry byproducts, olive oil byproducts, olive leaves, and alfalfa, all already available in large quantities, pending further detailed bioeconomic analysis. 

## Figures and Tables

**Table 1:** Herbs, spices, other plants and byproducts reviewed for immunomodulatory properties.

Herbs	Spices	Other Plants	Byproducts
Dill	Ginger	Alfalfa	Fruit pomace
Oregano	Coriander	Tea	Olive pomace
Skull cap	Galbanum	Turnip	
Ginkgo biloba	Sumac	Mushroom	
Sage	Black cumin	Catsclaw	
Fennel	Fenugreek	Glove artichoke	
Olive	Clove	Milk Thistle	
Hogweed	Chilli	Artemesia annua	
Purple coneflower		Milk vetch	
Thyme		Arfaj	
Chicory		Desert thorn	
		Spirulina	

## Data Availability

Not applicable.

## References

[1] Muaz K., Riaz M., Akhtar S., Park S., Ismail A. (2018). Antibiotic Residues in Chicken Meat: Global Prevalence, Threats, and Decontamination Strategies: A Review. J. Food Prot..

[2] Seidavi A., Zaker-Esteghamati H., Salem A.Z. (2020). A review on practical applications of *Citrus sinensis* by-products and waste in poultry feeding. Agrofor. Syst..

[3] Mehdi Y., Létourneau-Montminy M.P., Gaucher M.L., Chorfi Y., Suresh G., Rouissi T., Brar S.K., Côté C., Ramirez A.A., Godbout S. (2018). Use of antibiotics in broiler production: Global impacts and alternatives. Anim. Nutr..

[4] Pliego A.B., Tavakoli M., Khusro A., Seidavi A., Elghandour M.M., Salem A.Z., Rene Rivas-Caceres R. (2022). Beneficial and adverse effects of medicinal plants as feed supplements in poultry nutrition: A review. Anim. Bio..

[5] Ravindran V., Dryden G.M., Phillips C.J.C., Fuller M. (2023). Additives. Encyclopaedia of Animal Nutrition.

[6] FAO (2003). Regulation (EC) No 1831/2003 of the European Parliament and of The council of 22 September 2003 on Additives for Use in Animal Nutrition. Off. J. Europ. Union.

[7] Florez-Mendez J., Lopez J., Valencia G.A. (2022). Food Additives: Importance, Classification, and Adverse Reactions in Humans. Natural Additives in Foods.

[8] Hogeback J. What is the Difference between an Herb and a Spice. https://www.britannica.com/story/whats-the-difference-between-an-herb-and-a-spice.

[9] Placha I., Gai F., Pogány Simonová M. (2022). Natural feed additives in animal nutrition—Their potential as functional feed. Front. Vet. Sci..

[10] Nabavi S.M., Nabavi M.R., Loizzo R., Tundis R., Pandima Devi K., Sanches Silva A. (2020). Food Additives and Human Health.

[11] Valencia G.A. (2023). Natural Additives in Foods.

[12] Korver D.R. (2012). Implications of changing immune function through nutrition in poultry. Anim. Feed. Sci. Technol..

[13] Lauridsen C. (2019). From oxidative stress to inflammation: Redox balance and immune system. Poult. Sci..

[14] Hasted T.L., Sharif S., Boerlin P., Diarra M.S. (2021). Immunostimulatory potential of fruits and their extracts in poultry. Front. Immunol..

[15] Alloui M.N., Agabou A., Alloui N. (2014). Application of herbs and phytogenic feed additives in poultry production-A Review. Glob. J. Anim. Sci. Res..

[16] Kahkeshani N., Farzaei F., Fotouhi M., Alavi S.S., Bahramsoltani R., Naseri R., Bishayee A. (2019). Pharmacological effects of gallic acid in health and diseases: A mechanistic review. Iran. J. Basic Med. Sci..

[17] Craig W.J. (1999). Health-promoting properties of common herbs. Am. J. Clin. Nutr..

[18] Chen L., Cao H., Huang Q., Xiao J., Teng H. (2022). Absorption, metabolism and bioavailability of flavonoids: A review. Crit. Rev. Food Sci. Nutr..

[19] Shen N., Wang T., Gan Q., Liu S., Wang L., Jin B. (2022). Plant flavonoids: Classification, distribution, biosynthesis, and antioxidant activity. Food Chem..

[20] North M.K., DalleZotte A., Hoffman L.C. (2019). The use of dietary flavonoids in meat production: A review. Anim. Feed. Sci. Technol..

[21] Garg S.K., Shukla A., Choudhury S. (2019). Polyphenols and flavonoids. Nutraceuticals in Veterinary Medicine.

[22] Hosseinzade A., Sadeghi O., NaghdipourBiregani A., Soukhtehzari S., Brandt G.S., Esmaillzadeh A. (2019). Immunomodulatory effects of flavonoids: Possible induction of T CD4+ regulatory cells through suppression of mTOR pathway signaling activity. Front. Immunol..

[23] Saeed M., Naveed M., Arain M.A., Arif M., Abd El-Hack M.E., Alagawany M., Sun C. (2017). Quercetin: Nutritional and beneficial effects in poultry. World’s Poult. Sci. J..

[24] Shehata A.A., Yalçın S., Latorre J.D., Basiouni S., Attia Y.A., Abd El-Wahab A., Tellez-Isaias G. (2022). Probiotics, prebiotics, and phytogenic substances for optimizing gut health in poultry. Microorganisms.

[25] Catoni C., Schaefer H.M., Peters A. (2008). Fruit for Health: The Effect of Flavonoids on Humoral Immune Response and Food Selection in a Frugivorous Bird. Func. Ecol..

[26] Surai P.F., Kochish I.I., Fisinin V.I., Kidd M.T. (2019). Antioxidant defense systems and oxidative stress in poultry biology: An update. Antioxidants.

[27] Surai P.F., Kochish I.I., Romanov M.N., Griffin D.K. (2019). Nutritional modulation of the antioxidant capacities in poultry: The case of vitamin E. Poult. Sci..

[28] Bearth A., Cousin M.E., Siegrist M. (2014). The consumer’s perception of artificial food additives: Influences on acceptance, risk and benefit perceptions. Food Qual. Pref..

[29] Jiang J., Wu C., Gao H., Song J., Li H. (2010). Effects of *Astragalus* polysaccharides on immunologic function of erythrocyte in chickens infected with infectious bursa disease virus. Vaccine.

[30] Righi F., Pitino R., Manuelian C.L., Simoni M., Quarantelli A., De Marchi M., Tsiplakou E. (2021). Plant feed additives as natural alternatives to the use of synthetic antioxidant vitamins on poultry performances, health, and oxidative status: A review of the literature in the last 20 years. Antioxidants.

[31] Johnson-Dahl M.L., Zuidhof M.J., Korver D.R. (2017). The effect of maternal canthaxanthin supplementation and hen age on breeder performance, early chick traits, and indices of innate immune function. Poult. Sci..

[32] Nogareda C., Moreno J.A., Christou P. (2016). Carotenoid-enriched transgenic corn delivers bioavailable carotenoids to poultry and protects them against coccidiosis. Plant Biotechnol. J..

[33] Abd El-Hack M.E., Abdelnour S.A., Taha A.E., Khafaga A.F., Arif M., Ayasan T., Abdel-Daim M.M. (2020). Herbs as thermoregulatory agents in poultry: An overview. Sci. Total Environ..

[34] Azzam M.M., Jiang S.Q., Chen J.L., Lin X.J., Gou Z.Y., Fan Q.L., Jiang Z.Y. (2019). Effect of soybean isoflavones on growth performance, immune function, and viral protein 5 mRNA expression in broiler chickens challenged with infectious bursal disease virus. Animals.

[35] Jiang Z.Y., Jiang S.Q., Lin Y.C., Xi P.B., Yu D.Q., Wu T.X. (2007). Effects of soybean isoflavone on growth performance, meat quality, and antioxidation in male broilers. Poult. Sci..

[36] Zhang C., Zhao X.H., Yang L., Chen X.Y., Jiang R.S., Jin S.H., Geng Z.Y. (2017). Resveratrol alleviates heat stress-induced impairment of intestinal morphology, microflora, and barrier integrity in broilers. Poult. Sci..

[37] Wang C., Zhao F., Li Z., Jin X., Chen X., Geng Z., Hu H., Zhang C. (2021). Effects of Resveratrol on Growth Performance, Intestinal Development, and Antioxidant Status of Broilers under Heat Stress. Animals.

[38] Manna S.K., Mukhopadhyay A., Aggarwal B.B. (2000). Resveratrol suppresses TNF-α induced activation of nuclear transcription factors NF-κB, activator protein-1, and apoptosis: Potential role of reactive oxygen intermediates and lipid peroxidation. J. Immunol..

[39] He S., Chen L., He Y., Chen F., Ma Y., Xiao D., He J. (2020). Resveratrol alleviates heat stress-induced impairment of intestinal morphology, barrier integrity and inflammation in yellow-feather broilers. Anim. Prod. Sci..

[40] Pan M.H., Wu J.C., Ho C.T., Lai C.S. (2018). Antiobesity molecular mechanisms of action: Resveratrol and pterostilbene. Biofactors.

[41] Cai T.T., Ye X.L., Li R.R., Chen H., Wang Y.Y., Yong H.J., Ding D.F. (2020). Resveratrol modulates the gut microbiota and inflammation to protect against diabetic nephropathy in mice. Front. Pharmacol..

[42] Hammod A.J., Zeny Z.A.H., Mahdi A.S., Alfertosi K.A. (2021). Probiotic and humic acid as feed additives and their effects on productive and economic traits of broiler. Ind. J. Ecol..

[43] Chen C.H., Liu J.J., Lu F.J., Yang M.L., Lee Y., Huang T.S. (2002). The effect of humic acid on the adhesibility of neutrophils. Thromb. Res..

[44] Tohid T., Hasan G., Alireza T. (2010). Efficacy of mannanoligosaccharides and humate on immune response to Avian Influenza (H9) disease vaccination in broiler chickens. Vet. Res. Commun..

[45] Arif M., Alagawany M., Abd El-Hack M.E., Saeed M., Arain M.A., Elnesr S.S. (2019). Humic acid as a feed additive in poultry diets: A review. Iran. J. Vet. Res..

[46] Bornet F.R.J., Brouns F., Tashiro Y., Duvillier V. (2002). Nutritional aspects of short-chain fructooligosaccharides: Natural occurrence, chemistry, physiology and health implications. Dig. Liver Dis..

[47] Rafiei-Tari A., Karimi K., Hosseini S.A., Meimandipour A. (2016). Growth performance, carcass characteristics and serum biochemicals of Japanese quails fed with oat bran (*Avena sativa*) and dill seed (*Anethum graveolens*). Iran. J. Appl. Anim. Sci..

[48] Vispute M.M., Sharma D., Biswas A.K., Rokade J.J., Chapel A.T., Biswas A., Kapgate M.G. (2021). Dietary hemp (*Cannabis sativa* L) and dill seed (*Anethum graveolens*) improve physicochemical properties, oxidative stability, and sensory attributes of broiler meat. ACS Food Sci. Technol..

[49] Rahimian Y., Kheiri F., Alavi M., Aboozar M. (2017). Effect of using different levels of Dill seeds on performance, some blood biochemical and intestinal microbial population in Ross 308 broiler chicks. J. Med. Herbs.

[50] Zhao Q., Chen X.Y., Martin C. (2016). *Scutellaria baicalensis*, the golden herb from the garden of Chinese medicinal plants. Sci. Bull..

[51] Króliczewska B., Graczyk S., Króliczewski J., Pliszczak-Król A., Miśta D., Zawadzki W. (2017). Investigation of the immune effects of *Scutellariabaicalensis* on blood leukocytes and selected organs of the chicken’s lymphatic system. J. Anim. Sci. Biotechnol..

[52] Khafaga A.F., Bayad A.E. (2016). Impact of *Ginkgo biloba* extract on reproductive toxicity induced by single or repeated injection of cisplatin in adult male rats. Int. J. Pharmacol..

[53] Kim Y.J., Bostami A.B.M.R., Islam M.M., Mun H.S., Ko S.Y., Yang C.J. (2016). Effect of fermented *Ginkgo biloba* and *Camelia sinensis*-based probiotics on growth performance, immunity and cecal microbiology in broilers. Int. J. Poult. Sci..

[54] El Iraqi K.G., Abdelgawad E.M., Ibrahim H.M., El Sawe A.E. (2013). Effect of *Gingko Biloba*, dry peppermint and vitamin C as anti-stress on broiler welfare during summer heat stress. Glob. Vet..

[55] Liu X., Cao G., Wang Q., Yao X., Fang B. (2015). The effect of bacillus coagulans-fermented and nonfermented ginkgo biloba on the immunity status of broiler chickens. J. Anim. Sci..

[56] Rasouli B., Movahhedkhah S., Seidavi A., Haq Q.M.I., Kadim I., Laudadio V., Tufarelli V. (2020). Effect of sage (*Salvia officinalis* L) aqueous leaf extract on performance, blood constituents, immunity response and ileal microflora of broiler chickens. Agrofor. Syst..

[57] Farhadi M., Hedayati M., Manafi M., Khalaji S. (2020). Influence of using sage powder (*Salvia officinalis*) on performance, blood cells, immunity titers, biochemical parameters and small intestine morphology in broiler chickens. Iran. J. Appl. Anim. Sci..

[58] Safaei-Cherehh A., Rasouli B., Alaba P.A., Seidavi A. (2020). Effect of dietary *Foeniculum vulgare Mill* extract on growth performance, blood metabolites, immunity and ileal microflora in male broilers. Agrofor. Syst..

[59] Talhaoui N., Vezza T., Gómez-Caravaca A.M., Fernandez-Gutierrez A., Galvez J., Segura-Carretero A. (2016). Phenolic compounds and in vitro immunomodulatory properties of three Andalusian olive leaf extracts. J. Funct. Foods.

[60] Farràs M., Martinez-Gili L., Portune K., Arranz S., Frost G., Tondo M., Blanco-Vaca F. (2020). Modulation of the gut microbiota by olive oil phenolic compounds: Implications for lipid metabolism, immune system, and obesity. Nutrients.

[61] Millman J., Okamoto S., Kimura A., Uema T., Higa M., Yonamine M., Masuzaki H. (2020). Metabolically and immunologically beneficial impact of extra virgin olive and flaxseed oils on composition of gut microbiota in mice. Eur. J. Nutr..

[62] Pirman T., Rezar V., Vrecl M., Salobir J., Levart A. (2021). Effect of Olive Leaves or Marigold Petal Extract on Oxidative Stress, Gut Fermentative Activity, and Mucosa Morphology in Broiler Chickens Fed a Diet Rich in n-3 Polyunsaturated Fats. J. Poult. Sci..

[63] Espeso J., Isaza A., Lee J.Y., Sörensen P.M., Jurado P., Avena-Bustillos R de J., Olaizola M., Arboleya J.C. (2021). Olive leaf waste management. Front. Sustain. Food Syst..

[64] Vezza T., Algieri F., Rodríguez-Nogales A., Garrido-Mesa J., Utrilla M.P., Talhaoui N., Gálvez J. (2021). Immunomodulatory properties of *Olea europaea* leaf extract in intestinal inflammation. Mol. Nutr. Food Res..

[65] Bahadori Z., Esmaielzadeh L., Karimi-Torshizi M.A., Seidavi A., Olivares J., Rojas S., López S. (2017). The effect of earthworm (*Eisenia foetida*) meal with vermi-humus on growth performance, hematology, immunity, intestinal microbiota, carcass characteristics, and meat quality of broiler chickens. Livest. Sci..

[66] Jamshidparvar A., Javandel F., Seidavi A., Peña Blanco F., Martínez Marín A.L., Avilés Ramírez C., Núñez-Sánchez N. (2022). Effects of golpar (*Heracleum persicum Desf.*) and probiotics in drinking water on performance, carcass characteristics, organ weights, blood plasma constituents, and immunity of broilers. Environ. Sci. Pollut. Res..

[67] Javandel F., Nosrati M., Van den Hoven R., Seidavi A., Laudadio V., Tufarelli V. (2019). Effects of Hogweed (*Heracleum persicum*) powder, flavophospholipol, and probiotics as feed supplements on the performance, carcass and blood characteristics, intestinal microflora, and immune response in broilers. J. Poult. Sci..

[68] Kheiri F., Rahimian Y., Rafiee A. (2014). Effect of *Heracleum persicum* extract on performance and some haematological parameters in broiler chicks. Res. Opin. Anim. Vet. Sci..

[69] Nosrati M., Javandel F., Camacho L.M., Khusro A.M.E.E.R., Cipriano M., Seidavi A., Salem A.Z.M. (2017). The effects of antibiotic, probiotic, organic acid, vitamin C, and *Echinacea purpurea* extract on performance, carcass characteristics, blood chemistry, microbiota, and immunity of broiler chickens. J. Appl. Poult. Res..

[70] Movahhedkhah S., Rasouli B., Seidavi A., Mazzei D., Laudadio V., Tufarelli V. (2019). Summer Savory (*Satureja hortensis* L.) Extract as natural feed additive in broilers: Effects on growth, plasma constituents, immune response, and ileal microflora. Animals.

[71] Pournazari M., Qotbi A.A.A., Seidavi A., Corazzin M. (2017). Prebiotics, probiotics and thyme (*Thymus vulgaris*) for broilers: Performance, carcass traits and blood variables. Rev. Colomb. Cienc. Pec..

[72] Belali M., Seidavi A., Bouyeh M. (2021). Effects of short-term and combined use of thyme powder and aqueous extract on growth performance, carcass and organ characteristics, blood constituents, enzymes, immunity, intestinal morphology and fatty acid profile of breast meat in broilers. Large Anim. Rev..

[73] Fallah R., Mirzaei E. (2016). Effect of dietary inclusion of turmeric and thyme powders on performance, blood parameters and immune system of broiler chickens. J. Livestock Sci..

[74] Khoobani M., Hasheminezhad S.H., Javandel F., Nosrati M., Seidavi A., Kadim I.T., Laudadio V., Tufarelli V. (2019). Effects of dietary chicory (*Chicorium intybus* L.) and probiotic blend as natural feed additives on performance traits, blood biochemistry, and gut microbiota of broiler chickens. Antibiotics.

[75] Abd El-Hack M.E., Alagawany M., Shaheen H., Samak D., Othman S.I., Allam A.A., Taha A.E., Khafaga A.F., Arif M., Osman A. (2020). Ginger and its derivatives as promising alternatives to antibiotics in poultry feed. Animals.

[76] Qorbanpour M., Fahim T., Javandel F., Nosrati M., Paz E., Seidavi A., Tufarelli V. (2018). Effect of dietary ginger (*Zingiber officinale Roscoe*) and multi-strain probiotic on growth and carcass traits, blood biochemistry, immune responses and intestinal microflora in broiler chickens. Animals.

[77] Elmowalid G.A., Abd El-Hamid M.I., Abd El-Wahab A.M., Atta M., Abd El-Naser G., Attia A.M. (2019). Garlic and ginger extracts modulated broiler chicks innate immune responses and enhanced multidrug resistant Escherichia coli O78 clearance. Comp. Immunol. Microbiol. Infect. Dis..

[78] Abou-Elkhair R., Ahmed H., Selim S. (2014). Effects of Black Pepper (*Piper nigrum*), Turmeric Powder (*Curcuma longa*) and Coriander Seeds (*Coriandrum sativum*) and Their Combinations as Feed Additives on Growth Performance, Carcass Traits, Some Blood Parameters and Humoral Immune. Asian-Australas. J. Anim. Sci..

[79] Nadeem M., Anjum F.M., Khan M.I., Tehseen S., El-Ghorab A., Sultan J.I. (2013). Nutritional and medicinal aspects of coriander (*Coriandrum sativum* L.): A review. Brit. Food J..

[80] Naeemasa M., Qotbi A.A., Seidavi A., Norris D., Brown D., Ginindza M. (2014). Effects of coriander (*Coriandrum sativum* L) seed powder and extract on performance of broiler chickens. S. Afric. J. Anim. Sci..

[81] Hosseinzadeh H., AlawQotbi A.A., Seidavi A., Norris D., Brown D. (2014). Effects of different levels of coriander (*Coriandrum sativum*) seed powder and extract on serum biochemical parameters, microbiota, and immunity in broiler chicks. Sci. World J..

[82] Sarchahi A., Ghazvinian K., Kafshdoozan K., Jamshidi R. (2019). Effects of virginiamycin and galbanum (*Ferulagummosaboiss*) on performance, carcass traits, immune system and blood parameters of broiler chickens. Rev. Colomb. Cienc. Pec..

[83] Ahmadian A., Seidavi A., Phillips C.J.C. (2020). Growth and Carcass Composition, Haematology and Immunity of Broilers Supplemented with Sumac Berries (*Rhuscoriaria* L.) and Thyme (*Thymus vulgaris*). Animals.

[84] Azizi M., Passantino G., Akter Y., Javandel F., Seidavi A., Bahar B., Tufarelli V. (2020). Effect of sumac (*Rhus coriaria* L) seed powderon growth, carcass traits, blood parameters, immune system and selected ileal microorganisms of broilers. Vet. Ital..

[85] Forouzanfar F., Bazzaz B.S.F., Hosseinzadeh H. (2014). Black cumin (*Nigella sativa*) and its constituent (thymoquinone): A review on antimicrobial effects. Iran. J. Basic Med. Sci..

[86] Kumar P., Patra A.K., Samanta I., Pradhan S. (2014). Effect of black cumin seeds on growth performance, nutrient utilization, immunity, gut health and nitrogen excretion in broiler chickens. J. Sci. Food Agric..

[87] Laudadio V., Nasiri-Dehbaneh M., Bilal R.M., Qotbi A., Javandel F., Ebrahimi A., Tufarelli V. (2020). Effects of different levels of dietary black cumin (*Nigella sativa* L) and fenugreek (*Trigonella foenumgraecum* L) and their combination on productive traits, selected blood constituents, microbiota and immunity of broilers. Anim. Biotechnol..

[88] Farouk S.M., Abdel-Rahman H.G., Abdallah O.A., El-Behidy N.G. (2022). Comparative immunomodulatory efficacy of rosemary and fenugreek against *Escherichia coli* infection via suppression of inflammation and oxidative stress in broilers. Environ. Sci. Pollut. Res..

[89] Abouzar M., Rahimian Y. (2022). Response of broiler chicks fed by clove and cardamom alcoholic extracts. Braz. J. Hyg. Anim. Sanity.

[90] Kang C., Zhang Y., Zhu X., Liu K., Wang X., Chen M., Mi M. (2016). Healthy subjects differentially respond to dietary capsaicin correlating with specific gut enterotypes. J. Clin. Endocrinol. Metab..

[91] Mohamed S.H., Attia A.I., Reda F.M., Abd El-Hack M.E., Ismail I.E. (2021). Impacts of dietary supplementation of *Boswellia serrata* on growth, nutrients digestibility, immunity, antioxidant status, carcass traits and caecum microbiota of broilers. Ital. J. Anim. Sci..

[92] Kiczorowska B., Samolińska W., Al-Yasiry A.R.M., Kiczorowski P., Winiarska-Mieczan A. (2017). The natural feed additives as immunostimulants in monogastric animal nutrition–a review. Ann. Anim. Sci..

[93] Yıldız A.Ö., Şentürk E.T., Olgun O. (2020). Use of alfalfa meal in layer diets–A review. World’s Poult. Sci. J..

[94] Dong X.F., Gao W.W., Tong J.M., Jia H.Q., Sa R.N., Zhang Q. (2007). Effect of Polysavone (Alfalfa Extract) on Abdominal Fat Deposition and Immunity in Broiler Chickens. Poult. Sci..

[95] Pietrzak K., Grela E.R. (2015). Influence of alfalfa protein concentrate dietary supplementation on blood parameters of growing-finishing pigs. J. Vet. Res..

[96] (2021). Eghbaldost-Jadid R, Nosrati M, Rasouli B, Seidavi A, Phillips CJC The effects of turnip (*Brassica rapa*) extract on the growth performance and health of broilers. Animals.

[97] Jafarian-Dehkordi A., Zolfaghari B., Mirdamadi M. (2013). The effects of chloroform, ethyl acetate and methanolic extracts of *Brassica rapa* L on cell-mediated immune response in mice. Res. Pharmaceut. Sci..

[98] Willis W.L., Wall D.C., Isikhuemhen O.S., Jackson J.N., Ibrahim S., Hurley S.L., Anike F. (2013). Effect of level and type of mushroom on performance, blood parameters and natural coccidiosis infection in floor-reared broilers. Open Mycol. J..

[99] Kavyani A., Zare S.A., PorReza J., Jalali H.A.S.A. (2012). Evaluation of dried powder of mushroom (*Agaricusbisporus*) as an antibiotic growth promoter substitution on performance, carcass traits and humoral immune responses in broiler chickens. J. Med. Plants.

[100] Yang Y., Iji P.A., Choct M. (2009). Dietary modulation of gut microflora in broiler chickens: A review of the role of six kinds of alternatives to in-feed antibiotics. World’s Poult. Sci. J..

[101] Stoev S.D., Anguelov G., Ivanov I., Pavlo D. (2000). Influence of OA and extract of artichoke on the vaccinial immunity and health in broiler chicks. Exp. Toxicol. Pathol..

[102] Zaker-Esteghamati H., Seidavi A., Bouyeh M. (2020). A review on the effect of *Silybum marianum* and its derivatives on broilers under healthy and aflatoxicosis conditions: Part 1: Performance, carcass and meat characteristics, and intestinal microflora. World’s Poult. Sci. J..

[103] Chand N., Din Muhammad F., Durrani R., Subhan Qureshi M., Shahibzada S.U. (2011). Protective effect of milk thistle (*Silybum marianum*) against Aflatoxin B1 in broiler chicks. J. Anim. Sci..

[104] Gazak R., Walterova D., Kren V. (2007). Silybin and silymarin-new and emerging applications in medicine. Curr. Med. Chem..

[105] Feshanghchi M., Baghban-Kanani P., Kashefi-Motlagh B., Adib F., Azimi-Youvalari S., Hosseintabar-Ghasemabad B., Tufarelli V. (2022). Milk thistle (*Silybum marianum*), marine algae (*Spirulina platensis*) and toxin binder powders in the diets of broiler chickens exposed to aflatoxin-b1: Growth performance, humoral immune response and cecal microbiota. Agriculture.

[106] Baldi A., Dixit V.K. (2008). Yield enhancement strategies for artemisinin production by suspension cultures of *Artemisia annu*. Bioresour. Technol..

[107] Baghban-Kanani P., Hosseintabar-Ghasemabad B., Azimi-Youvalari S., Seidavi A., Ragni M., Laudadio V., Tufarelli V. (2019). Effects of using *Artemisia annua* leaves, probiotic blend, and organic acids on performance, egg quality, blood biochemistry, and antioxidant status of laying hens. J. Poult. Sci..

[108] Wan X.L., Niu Y., Zheng X.C., Huang Q., Su W.P., Zhang J.F., Wang T. (2016). Antioxidant capacities of *Artemisia annua* L leaves and enzymatically treated *Artemisia annua* L. in vitro and in broilers. Anim. Feed. Sci. Technol..

[109] Xi N., Kang J., Hao L., Li R., Bao Y., Shi W. (2014). Effects of ultrafine powder of the stem and leaf of *Astragalus* on immunity in chickens. Ital. J. Anim. Sci..

[110] Farag M.R., Alagawany M. (2019). The role of *Astragalus membranaceus* as immunomodulator in poultry. World’s Poult. Sci. J..

[111] Shan C., Sun B., Dalloul R.A., Zhai Z., Sun P., Li M., Luan W. (2019). Effect of the oral administration of *Astragalus* polysaccharides on jejunum mucosal immunity in chickens vaccinated against Newcastle disease. Microb. Path..

[112] Albarrak S.M. (2021). Supplementation with the methanolic extract of *Lycium shawii* or *Rhanterium epapposum* enhances immune responses of broilers. Adv. Anim. Vet. Sci..

[113] Tufarelli V., Baghban-Kanani P., Azimi-Youvalari S., Hosseintabar-Ghasemabad B., Slozhenkina M., Gorlov I., Laudadio V. (2021). Effects of horsetail (*Equisetum arvense*) and spirulina (*Spirulina platensis*) dietary supplementation on laying hens’ productivity and oxidative status. Animals.

[114] Seidavi A., Zaker-Esteghamati H., Scanes C.G., Simpson B.K., Aryee A.N., Toldra F. (2019). Poultry byproducts. Byproducts from Agriculture and Fisheries. Adding Value for Food, Feed, Pharma, and Fuels.

[115] Ragab M.S., Hassan H.A. (2007). Effects of using dried Egyptian clover and orange peels as natural feed additives on egg production egg quality and immune response of laying hens. Fayoum J. Agric. Res. Devel..

[116] Di Majo D., Giammanco M., La Guardia M., Tripoli E., Giammanco S., Finotti E. (2005). Flavanones in Citrus fruit: Structure antioxidant activity relationships. Food Res. Int..

[117] Ebrahimi A., Santini A., Alise M., Pourhossein Z., Miraalami N., Seidavi A. (2015). Effect of dried *Citrus sinensis* peel on gastrointestinal microbiota and immune system traits of broiler chickens. Ital. J. Anim. Sci..

[118] Pourhossein Z., Qotbi A.A.A., Seidavi A., Laudadio V., Centoducati G., Tufarelli V. (2015). Effect of different levels of dietary sweet orange (*Citrus sinensis*) peel extract on humoral immune system responses in broiler chickens. Anim. Sci. J..

[119] Pourhossein Z., Qotbi A.A.A., Seidavi A., Laudadio V., Mazzei D., Tufarelli V. (2019). Feeding of dried sweet orange (*Citrus sinensis*) peel on humoral immune response of broiler chickens. Int. J. Recycl. Organ. Waste Agric..

[120] De Oliveira C.O., Roll A.A.P., Xavier E.G. (2021). Olive pomace for the feeding of commercial poultry: Effects on performance, meat and eggs quality, haematological parameters, microbiota and immunity. World’s Poult. Sci. J..

[121] Sateri S., Seidavi A., Bouyeh M. (2014). Effects of olive pulp and multi enzyme, thymus on liver, spleen and bursa of Fabricius of broiler chickens. S. Afric. J. Anim. Sci..

[122] Bahadori M.B., Dinparast L., Zengin G. (2016). The genus *Heracleum*: A comprehensive review on its phytochemistry, pharmacology, and ethnobotanical values as a useful herb. Comp. Rev. Food Sci. Food Saf..

[123] Popović M., Grdiša M., Hrženjak T.M. (2005). Glycolipoprotein G-90 obtained from the earthworm *Eisenia foetida* exerts antibacterial activity. Veterinarskiarhiv.

[124] Chashmidari Y., Esmaielzadeh L., Karimi-Torshizi M.A., Seidavi A., Da Silva Araujo C.S., Araujo L.F. (2021). Feed supplementation with vermi-humus and earthworm (*Eisenia foetida*) powder on broiler productivity. Ital. J. Anim. Sci..

[125] Coman V., Teleky B.-E., Mitrea L., Martău G.A., Szabo K., Călinoiu L.F., Vodnar D.C., Fidel T. (2020). Bioactive potential of fruit and vegetable wastes. Advances in Food and Nutrition Research.

[126] Cea Pavez I., Lozano-Sánchez J., Borrás-Linares I., Nuñez H., Robert P., Segura-Carretero A. (2019). Obtaining an Extract Rich in Phenolic Compounds from Olive Pomace by Pressurized Liquid Extraction. Molecules.

[127] Hansen C. (2022). Environmental Impact of Olive Oil Processing.

[128] Aydogan I., Yildirim E., Kurum A., Bolat D., Cinar M., Basalan M., Yigit A. (2022). The Effect of Dietary Garlic (*Allium Sativum*), Black Cumin (*Nigella Sativa*) and Their Combination on Performance, Intestine Morphometry, Serum Biochemistry and Antioxidant Status of Broiler Chickens. Braz. J. Poult. Sci..

[129] Mordor Intelligence Poultry Feed Market, Growth, Trends and Forecasts (2023–2028). Poultry Feed Market Size & Share Analysis—Industry Research Report—Growth Trends..

[130] di Giacomo G., Romano P. (2022). Evolution of the Olive Oil Industry along the Entire Production Chain and Related Waste Management. Energies.

[131] Abdelli N., Solà-Oriol D., Pérez J.F. (2021). Phytogenic Feed Additives in Poultry: Achievements, Prospective and Challenges. Animals.

